# What’s the difference between lung adenocarcinoma and lung squamous cell carcinoma? Evidence from a retrospective analysis in a cohort of Chinese patients

**DOI:** 10.3389/fendo.2022.947443

**Published:** 2022-08-29

**Authors:** Wen Wang, Hui Liu, Guoli Li

**Affiliations:** ^1^ Department of Cardio-Thoracic Surgery, Hunan Provincial People’s Hospital (The First-Affiliated Hospital of Hunan Normal University), Changsha, China; ^2^ Department of Nephrology and Laboratory of Kidney Disease, Hunan Provincial People’s Hospital (The First-Affiliated Hospital of Hunan Normal University), Changsha, China; ^3^ Changsha Clinical Research Center for Kidney Disease, Changsha, China; ^4^ Hunan Clinical Research Center for Chronic Kidney Disease, Changsha, China

**Keywords:** lung adenocarcinoma, lung squamous cell carcinoma, clinicopathologic characteristics, radiological features, laboratory data

## Abstract

**Background:**

Lung adenocarcinoma (LUAD) and lung squamous cell carcinoma (LUSC) are the two most common subtypes of lung cancer. Previously, they were categorized into one histological subtype known as non-small cell lung cancer (NSCLC) and often treated similarly. However, increasing evidence suggested that LUAD and LUSC should be classified and treated as different cancers. But yet, detailed differences in clinical features between LUAD and LUSC have not been well described.

**Methods:**

A cohort of 142 Chinese patients with 111 LUAD and 31 LUSC cases were consecutively enrolled from April 2019 to October 2020 in Hunan Provincial People’s Hospital. The clinical features of the patients were retrospectively analyzed and compared in the terms of general information, clinicopathologic characteristics, imaging findings and laboratory data.

**Results:**

In comparison with LUAD, LUSC patients had a significantly higher proportion of males, smokers, drinkers, higher-stage cases. The mean tumor size in LUSC patients was significantly larger than that in LUAD patients. Compared with LUAD patients, more of patients with LUSC had cough, fever and abundant sputum symptoms. Besides that, more bacterial infections and fungal infections were found in LUSC patients than that in LUAD patients. Imaging data shows that ground-glass opacity and patchy shadows in radiological films were more frequent in LUAD patients than that in LUSC patients. In addition to initial laboratory data, LUSC patients had higher levels of leukocytes, platelets, and creatinine that of LUAD patients.

**Conclusions:**

Together, these results suggested that there exist distinct differences between LUAD and LUSC subtypes; LUSC may be a more malignant type in comparison with LUAD. Our findings may have potential implications in clinical settings. However, further multicenter studies are needed to validate these findings in a larger sample size.

## Introduction

According to the newest update on the global cancer burden, lung cancer remained the leading cause of cancer-related death worldwide (1). Lung cancer is a highly heterogenous disease with wide-range of clinicopathological and molecular features (2). Within the classifications of lung cancer, non-small-cell lung cancer (NSCLC) accounts for approximately 85% of all cases. NSCLC can further subdivide into two most common subtypes, lung adenocarcinoma (LUAD) and lung squamous cell carcinoma (LUSC), representing 50–60% and 20–30% of total NSCLC cases, respectively (2, 3). Recent years has seen a rapid progress in development of personalized therapy in lung cancer, which lead to great benefit to thousands of patients (4). To further improve the treatment and prevention, a better understanding of the clinical features of LUAD and LUSC is needed.

It is widely believed that LUAD and LUSC are not only tumors with different histologic types, but also tumors with different biological signatures and clinical implications (5, 6). Previous studies suggested that LUAD and LUSC may derived from different epithelial cells, express different cell markers and have different genomic profiles (6–9). And a significant difference was found in their prognosis (10–12). However, for a long time in the past, they were often treated similarly (5). One of the main reasons is that a comprehensive understanding of their distinct clinical characteristics and behaviors are still largely unknown.

In the present study, with the aim to deepen the understanding of the difference between LUAD and LUSC, we conducted a comprehensive analysis to compare the basic clinical information, tumor characteristics, radiological features and laboratory data of patients between LUAD and LUSC. Our results highlighted some distinct features of these two cancer types with potential diagnostic and prognostic values which may be warranted in clinical settings in the future.

## Material and methods

### Patient enrollment

A total of 142 Chinese patients with stage I–IV NSCLC were consecutively collected in our hospital from April 2019 to October 2020, including 111 patients with LUAD and 31 patients with LUSC. We created this cohort by applying the following inclusion criteria: (1) aged 18-85; (2) with definite histological subtype results; (3) receiving surgery due to lung cancer in our hospital. Patients with adenosquamous carcinoma were excluded from this study, which accounted for less than 2% of the total NSCLC cases. This study was approved by the Ethics Committee of Hunan Provincial People’s Hospital. All participants have signed the informed consent.

The data at the time of diagnosis of all the patients were extracted from the medical record system. The following items were included: histological type, age, gender, smoking history, alcohol consumption, body mass index (BMI) education level, T classification, N classification, M classification, clinical stage, tumor size, tumor location, main symptoms (cough, fever, dyspnea, chest distress, headache, abundant sputum and swollen lymph nodes), the status of pathogenic microbial infection at lung when diagnosed (bacterial and fungal infection), analysis of blood biochemistry and cells (leukocytes, lymphocytes, platelets, red blood cells, hemoglobin and creatinine) and imaging data before surgery (ground-glass opacity, pleural effusion, patchy shadows, sign of air bronchogram, signs of boundary feature and sign of the cord). Lung cancer histologic confirmation was made according to the 2015 World Health Organization (WHO) classification guidelines (3). Patients were pathologically staged based on the eighth edition TNM classification (13, 14). BMI was calculated using the standard BMI formula: BMI = weight (kg)/[height (m)]^2^ and classified into 4 categories according to the WHO international classification: underweight (BMI < 18.5), normal weight (18.5 ≤ BMI < 25), overweight (25 ≤BMI < 30) and obese (≥30) (15).

### Statistical analysis

The associations of all above items between LUAD and LUSC groups were statistically analyzed. Student’s t-test was used to evaluate the continuous variable, and Fisher’s exact test was used to evaluate the categorical variables. All *p-*values were two-sided, and *p* < 0.05 was considered as statistically significant difference.

## Results

A total of 142 lung cancer cases were enrolled for analysis, including 111 LUAD cases and 31 LUSC cases. **Table 1** shows the baseline demographic and clinicopathological characteristics of the patients. We can see that the mean age, BMI and education level of patients with LUAD was comparable to that of patients with LUSC (*p=*0.904, *p=*0.713 and *p=*0.822, respectively). The gender distribution differed between the groups (*p* < 0.001). There were 50 (45.0%) males and 61 (55.0%) females in the LUAD group; while 30 (96.8%) males and 1 (3.2%) female in the LUSC group. The LUSC group had more smokers (74.2% vs 25.2%, *p* < 0.001) and drinkers (32.2% vs 10.8%, *p* =0.013) than that in the LUAD group. Additionally, different distributions were also seen in clinical TNM stage. LUSC was associated with a relatively higher stage (more cases in the stage II-IV; stage I: 25.8%, stage II: 38.7%, stage III: 29.0%, stage IV: 3.2%) in comparison with LUAD (stage I: 73.0%, stage II: 8.1%, stage III:12.6%, stage IV: 2.7%) (*p* < 0.001, **Table 1**). The tumor size (represented by major diameter) in the radiographic films was larger in the LUSC patients (4.3 cm vs 2.1 cm, *p* < 0.001) compared with that in the LUAD patients, and this difference was consistently observed in the resected tumors (3.5 cm vs 2.0 cm, *p* < 0.001). These characteristics implied that LUSC is associated with a higher malignancy in comparison with LUAD at the time of diagnosis.

**Table 1:** Demographic and tumour characteristics of patients.

Characteristics	LUAD (%)	LUSC (%)	P-value
Total	111 (100)	31 (100)	
Age			
Mean (range)	59.5 (35-83)	59.2 (27-72)	0.904^a^
<40	2 (1.8)	1 (3.2)	
40–60	55 (49.5)	15 (48.4)	
>60	54 (48.6)	15 (48.4)	
BMI (kg/m^2^)^b^			
Mean (range)	23.1 (15.1-32.9)	22.3 (16.7-27.9)	0.713^a^
Underweight	3 (2.7)	1 (3.2)	
Normal weight	68 (61.3)	21 (67.7)	
Overweight	18 (16.2)	4 (12.9)	
Obese	2 (1.8)	0 (0.0)	
Unknown	20 (18.0)	5 (16.1)	
Education^c^			0.822^d^
Primary	20 (18.0)	7 (22.6)	
Lower	59 (53.2)	17 (54.8)	
Intermediate	26 (23.4)	5 (16.1)	
Higher	6 (5.4)	2 (6.5)	
Gender			**<0.001^d^**
Male	50 (45.0)	30 (96.8)	
Female	61 (55.0)	1 (3.2)	
Smoking history			**<0.001^d^**
Never	80 (72.1)	5 (16.1)	
Ever	28 (25.2)	23 (74.2)	
Unknown	3 (2.7)	3 (9.7)	
Alcohol consumption			**0.013^d^**
Never	99 (89.2)	21 (67.7)	
Light	5 (4.5)	5 (16.1)	
Heavy	7 (6.3)	5 (16.1)	
Clinical T			**<0.001^d^**
T0, T1	79 (71.2)	6 (19.4)	
T2, T3, T4	31 (27.9)	24 (77.4)	
Unknown	1 (0.9)	1 (3.2)	
Clinical N			**0.001^d^**
N0	87 (78.4)	16 (51.6)	
N1, N2, N3	19 (17.1)	14 (45.2)	
Unknown	5 (4.5)	1 (3.2)	
Clinical M			0.303^d^
M0	107 (96.4)	28 (90.3)	
M1a, M1b, M1c	3 (2.7)	2 (6.5)	
Unknown	1 (0.9)	1 (3.2)	
Clinical stage			**<0.001^d^**
I	81 (73.0)	8 (25.8)	
II	9 (8.1)	12 (38.7)	
III	14 (12.6)	9 (29.0)	
IV	3 (2.7)	1 (3.2)	
Unknown	4 (3.6)	1 (3.2)	
Tumor size (cm)			
Mean (range) in actual tumor	2.0 (0.4-8.0)	3.5 (0.9-7.0)	**<0.001^a^**
Mean (range) in radiographic film	2.1 (0.6-7.0)	4.3 (1.3-8.1)	**<0.001^a^**
Tumor location			0.464^d^
Left	41 (36.9)	15 (48.4)	
Right	69 (62.2)	16 (51.6)	
Bilateral	1 (0.9)	0 (0.0)	

^a^ Student’s t-test; ^b^ Underweight (BMI < 18.5), normal weight (18.5 ≤ BMI < 25), overweight (25 ≤ BMI < 30) and obese (BMI ≥ 30); ^c^ Primary: primary education; lower: lower or intermediate general education, or lower vocational education; intermediate: intermediate vocational education or higher general education; higher: higher vocational education or university; ^d^ Pearson’s chi-squared test. ; P values in bold font indicates significant <0.05.

Next, we compared the differences of typical symptoms between the two subtypes of NSCLC. As **Table 2** shown, LUSC patients had a significantly higher proportion of cough (77.4% vs 29.7%, *p* < 0.001), fever (12.9% vs 0.9%, *p* = 0.001), and abundant sputum (54.8% vs 11.7%, *p* < 0.001) than LUAD. There were no significant differences in breathing difficulty, chest tightness, sore throat, fatigue, headache, nausea and vomiting, diarrhea and swollen lymph nodes. Additionally, more patients with LUSC were associated bacterial infection (12.9% vs 2.7%, *p* < 0.001) or fungal infection (16.1% vs 1.8%, *p* < 0.001) at the time of diagnosis compared with that in patients with LUAD.

**Table 2:** Main symptoms of the patients.

Symptoms	LUAD (%)	LUSC (%)	P-value^a^
Total	111 (100)	31 (100)	
Cough			**<0.001**
Yes	33 (29.7)	24 (77.4)	
No	78 (70.3)	7 (22.6)	
Fever			**0.001**
Yes	1 (0.9)	4 (12.9)	
No	110 (99.1)	27 (87.1)	
Breathing difficulty			0.206
Yes	12 (10.8)	6 (19.4)	
No	99 (89.2)	25 (80.6)	
Chest tightness			0.167
Yes	14 (12.6)	7 (22.6)	
No	97 (87.4)	24 (77.4)	
Sore throat			0.331
Yes	1 (0.9)	1 (3.2)	
No	110 (99.1)	30 (96.8)	
Fatigue			0.823
Yes	6 (5.4)	2 (6.5)	
No	105 (94.6)	29 (93.5)	
Headache			0.754
Yes	5 (4.5)	1 (3.2)	
No	106 (95.5)	30 (96.8)	
Abundant sputum			**<0.001**
Yes	13 (11.7)	17 (54.8)	
No	98 (88.3)	14 (45.2)	
Nausea and vomiting			0.459
Yes	2 (1.8)	0 (0.0)	
No	109 (98.2)	30 (96.8)	
Diarrhea			0.626
Yes	2 (1.8)	1 (3.2)	
No	109 (98.2)	30 (96.8)	
Swollen lymph nodes			0.139
Yes	92 (82.9)	29 (93.5)	
No	19 (17.1)	2 (6.5)	
Bacterial infections			**<0.001**
Yes	3 (2.7)	4 (12.9)	
No	35 (31.5)	20 (64.5)	
Unknown	73 (65.8)	7 (22.6)	
Fungal infection			**<0.001**
Yes	2 (1.8)	5 (16.1)	
No	36 (32.4)	18 (58.1)	
Unknown	73 (65.8)	8 (25.8)	

a Pearson’s chi-squared test ; P values in bold font indicates significant <0.05.

Further, the radiological features were compared between two subtypes of NSCLC (**Table 3**). No differences were found in the signs of boundary feature, air bronchogram, the cord and pleural effusion. However, LUAD patients showed higher ratios of ground-glass opacity (38.7% vs 0%, *p* < 0.001) and patchy shadows (96.4% vs 83.9%, *p* < 0.001) than that in LUSC patients.

**Table 3:** Imaging data of the patients.

Features	LUAD (%)	LUSC (%)	P-value^a^
Total	111 (100)	31 (100)	
Ground-glass opacity			**<0.001**
Yes	43 (38.7)	0 (0.0)	
No	68 (61.3)	31 (100.0)	
Boundary feature			0.061
Yes	108 (97.3)	30 (96.8)	
No	0 (0)	1 (3.2)	
Unknown	3 (2.7)	0 (0.0)	
Patchy shadows			**<0.001**
Yes	107 (96.4)	26 (83.9)	
No	1 (0.9)	5 (16.1)	
Unknown	3 (2.7)	0 (0.0)	
The cord sign			0.473
Yes	60 (54.1)	19 (61.3)	
No	51 (45.9)	12 (38.7)	
Air bronchogram sign			0.436
Yes	19 (17.1)	7 (22.6)	
No	92 (82.9)	23 (74.2)	
Unknown	0 (0)	1 (3.2)	
Pleural effusion			0.876
Yes	3 (2.7)	1 (3.2)	
No	108 (97.3)	30 (96.8)	

a Pearson’s chi-squared test; P values in bold font indicates significant <0.05.

**Figure 1:**
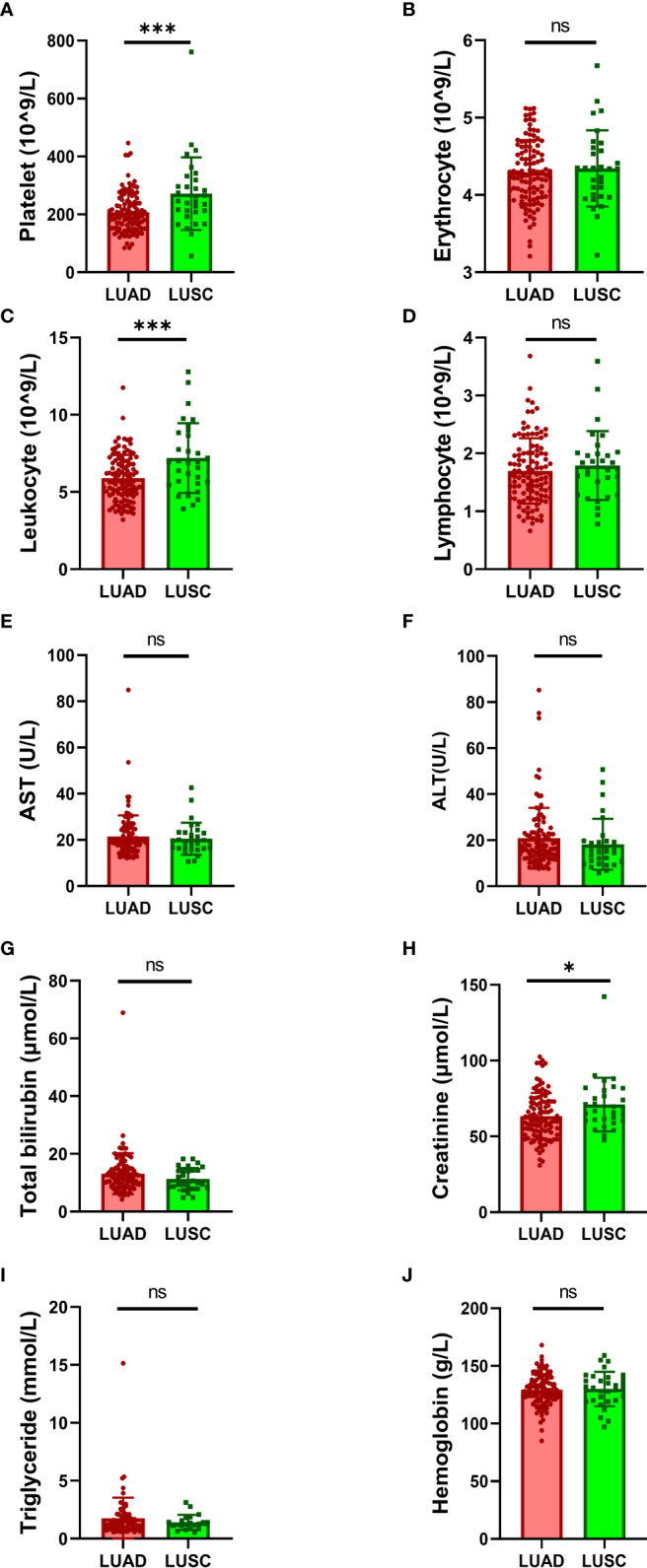
Comparison of laboratory data between LUAD and LUSC. Comparison of the number of blood cells: **(A)** platelets, **(B)** erythrocytes, **(C)** leukocytes and **(D)** lymphocytes. Comparison of the level of physiological indexes: **(E)** AST, **(F)** ALT, **(G)** total bilirubin, **(H)** creatinine, **(I)** triglyceride and **(J)** hemoglobin. AST, aspartate transaminase; ALT, alanine aminotransferase. Data were analyzed by the 2-tailed Student’s t test. *P < 0.05, ***P < 0.001; ns, not significant (p -value > 0.05).

Moreover, the laboratory data were also collected and analyzed in this study. We found that larger number of platelets (**Figure 1A**) and leukocytes (**Figure 1C**) in the blood of LUSC patients than that in LUAD patients. And higher level of creatinine in the blood of LUSC patients than that in LUAD patients was also found (**Figure 1H**). No significant differences were found between LUAD and LUSC subtypes in other blood indicators, including the number of erythrocytes (**Figure 1B**) and lymphocytes (**Figure 1D**), the level of aspartate transaminase (AST) (**Figure 1E**), alanine aminotransferase (ALT) (**Figure 1F**), total bilirubin (**Figure 1G**), triglyceride (**Figure 1I**) and hemoglobin (**Figure 1J**).

Additionally, no significant difference was found between LUAD and LUSC subtypes in history of other diseases, including other tumors, hypertension, hyperlipidemia, diabetes, cardiovascular diseases and cerebrovascular diseases (**Table 4**).

**Table 4:** History of other diseases of the patients.

Diseases	LUAD (%)	LUSC (%)	P-value^a^
Total	111 (100)	31 (100)	
Other tumors			0.511
Yes	7 (6.3)	1 (3.2)	
No	104 (93.7)	30 (96.8)	
Hypertension			0.372
Yes	30 (27.0)	6 (19.4)	
No	80 (72.1)	25 (80.6)	
Unknown	1 (0.9)	0 (0.0)	
Hyperlipidemia			0.505
Yes	7 (6.3)	1 (3.2)	
No	103 (92.8)	30 (96.8)	
Unknown	1 (0.9)	0 (0.0)	
Diabetes			0.192
Yes	12 (10.8)	1 (3.2)	
No	98 (88.3)	30 (96.8)	
Unknown	1 (0.9)	0 (0.0)	
Cardiovascular diseases			0.986
Yes	7 (6.3)	2 (6.5)	
No	103 (92.8)	29 (93.5)	
Unknown	1 (0.9)	0 (0.0)	
Cerebrovascular disease			0.748
Yes	5 (4.5)	1 (3.2)	
No	105 (94.6)	30	
Unknown	1 (0.9)	0 (0.0)	

a Pearson’s chi-squared test.

## Discussion

In this retrospective study, we investigated the clinical features in a cohort of 142 Chinese patients with NSCLC. Strikingly, we found that LUAD and LUSC subtypes have distinct characteristics in terms of gender, smoking history, alcohol consumption, clinical stage, tumor size, typical symptoms, microbial infection, radiological signs, and blood indicators, indicating that LUAD and LUSC may be separate clinical entities. To our best knowledge, this is the first study comprehensively describing the differences between LUAD and LUSC in Chinese population.

NSCLC is a very complex and heterogeneous disease (16). Different clinical and histopathological features, as well as molecular futures have been found between LUAD and LUSC (7, 9–11, 17–19). In consistency with previous reports (10, 11, 19), we found that there were more males, more smokers, more cases with high clinical stages and larger tumor size in LUSC than that in LUAD. In addition, we also found that patients with LUSC have more alcohol consumption, more frequent cough, fever and microbial infection than that in patients with LUAD. Furthermore, larger number of platelets and leukocytes and higher level of creatinine were also found in the blood of LUSC patients that that in LUAD patients. In all, our and other group’s findings suggested that LUSC may be a more malignant type in comparison with LUAD.

Previously, several studies have investigated the association between platelet count and prognosis in lung cancer (20–24). However, no study has reported that difference of platelet counts between LUSC and LUAD so far. To the best of our knowledge, this is the first study reporting that platelets was significantly higher in LUSC patients than that in LUAD patients. Platelets have been shown to play important roles in almost all steps of tumorigenesis such as angiogenesis, cell proliferation, cell invasiveness, and metastasis (25). These findings indicated that platelets count may be a useful biomarker for NSCLC diagnosis and prognosis.

Links between infection, inflammation and lung cancer has been fully defined and the mechanism has been established that systemic inflammatory pathways as effect of microbial persistence in the lung can secondarily promote the development of lung carcinogenesis (26). In the present study, we showed that patients with LUSC have more frequent cough, fever, microbial infection and larger number of leukocytes than that in patients with LUAD. Our results suggested that infection and inflammation may be more involved in LUSC than that in LUAD. However, further study is needed to explore these potential associations and underlying mechanisms.

It is important to note that this work has some limitations. First, this is a single-center study with a small sample size. Moreover, possibly due to the small sample size, we failed to discover any useful tool for predicting the classification of NSCLC before surgery. Using all the associated features we found, it is still impossible to establish a model for predicting the one-year survival in multivariate analysis. As the follow-up data was lacking, survival analysis was not performed in this study. So, the risk factors for overall survival of patients with LUAD and LUSC is still unknown. Another important future direction following this work is to collected more data to gain a better understanding of the outcomes of patients with different NSCLC subtypes.

## Conclusion

Taken together, LUAD and LUSC are subtypes of NSCLC with distinct characteristics in terms of gender composition, smoking or drinking habit, clinical stages, tumor size, typical symptoms, microbial infection, radiological signs, and laboratory values. Our findings suggested that a more detailed view on these two different subtypes is needed in clinical practices.

## Data availability statement

The raw data supporting the conclusions of this article will be made available by the authors, without undue reservation.

## Ethics statement

The studies involving human participants were reviewed and approved by This study was approved by the Ethics Committee of Hunan Provincial People’s Hospital. The patients/participants provided their written informed consent to participate in this study.

## Author contributions

WW and GL designed the study, WW and HL contributed collecting, analyzing and interpreting the data, WW and GL provided funding. GL wrote the manuscript. All authors read and approved the final manuscript.

## Funding

This project was supported by the Hunan Province Office of Education (No. 21B0052), Scientific Research Projects of the Health Commission of Hunan Province (No. 202203050006), Changsha Natural Science Foundation project (No. kq2202439) and Open Research Fund of the State Key Laboratory of Environmental Chemistry and Ecotoxicology (No. KF-2021-18).

## Conflict of interest

The authors declare that the research was conducted in the absence of any commercial or financial relationships that could be construed as a potential conflict of interest.

## Publisher’s note

All claims expressed in this article are solely those of the authors and do not necessarily represent those of their affiliated organizations, or those of the publisher, the editors and the reviewers. Any product that may be evaluated in this article, or claim that may be made by its manufacturer, is not guaranteed or endorsed by the publisher.

## References

[1] Sung H Ferlay J Siegel RL Laversanne M Soerjomataram I Jemal A. Global cancer statistics 2020: GLOBOCAN estimates of incidence and mortality worldwide for 36 cancers in 185 countries. CA Cancer J Clin (2021) 71:209–49. doi: 10.3322/caac.21660 33538338

[2] Thai AA Solomon BJ Sequist LV Gainor JF Heist RS. Lung cancer. Lancet (2021) 398:535–54. doi: 10.1016/S0140-6736(21)00312-3 34273294

[3] Travis WD Brambilla E Nicholson AG Yatabe Y Austin JHM Beasley MB. The 2015 world health organization classification of lung tumors: Impact of genetic, clinical and radiologic advances since the 2004 classification. J Thorac Oncol (2015) 10:1243–60. doi: 10.1097/JTO.0000000000000630 26291008

[4] Wang M Herbst RS Boshoff C. Toward personalized treatment approaches for non-small-cell lung cancer. Nat Med (2021) 27:1345–56. doi: 10.1038/s41591-021-01450-2 34385702

[5] Herbst RS Morgensztern D Boshoff C. The biology and management of non-small cell lung cancer. Nature (2018) 553:446–54. doi: 10.1038/nature25183 29364287

[6] Relli V Trerotola M Guerra E Alberti S. Abandoning the notion of non-small cell lung cancer. Trends Mol Med (2019) 25:585–94. doi: 10.1016/j.molmed.2019.04.012 31155338

[7] Campbell JD Alexandrov A Kim J Wala J Berger AH Pedamallu CS. Distinct patterns of somatic genome alterations in lung adenocarcinomas and squamous cell carcinomas. Nat Genet (2016) 48:607–16. doi: 10.1038/ng.3564 PMC4884143 27158780

[8] Anusewicz D Orzechowska M Bednarek AK. Lung squamous cell carcinoma and lung adenocarcinoma differential gene expression regulation through pathways of notch, hedgehog, wnt, and ErbB signalling. Sci Rep (2020) 10:21128. doi: 10.1038/s41598-020-77284-8 33273537 PMC7713208

[9] Chen JW Dhahbi J. Lung adenocarcinoma and lung squamous cell carcinoma cancer classification, biomarker identification, and gene expression analysis using overlapping feature selection methods. Sci Rep (2021) 11:13323. doi: 10.1038/s41598-021-92725-8 34172784 PMC8233431

[10] Kawase A Yoshida J Ishii G Nakao M Aokage K Hishida T. Differences between squamous cell carcinoma and adenocarcinoma of the lung: Are adenocarcinoma and squamous cell carcinoma prognostically equal? Japan J Clin Oncol (2012) 42:189–95. doi: 10.1093/jjco/hyr188 22210923

[11] Nakamura H Sakai H Kimura H Miyazawa T Marushima H Saji H. Difference in postsurgical prognostic factors between lung adenocarcinoma and squamous cell carcinoma. Ann Thorac Cardiovasc Surg (2017) 23:291–7. doi: 10.5761/atcs.oa.17-00020 PMC5738450 28966230

[12] Meng F Zhang L Ren Y Ma Q. The genomic alterations of lung adenocarcinoma and lung squamous cell carcinoma can explain the differences of their overall survival rates. J Cell Physiol (2019) 234:10918–25. doi: 10.1002/jcp.27917 30549039

[13] Detterbeck FC Boffa DJ Kim AW Tanoue LT. The eighth edition lung cancer stage classification. Chest (2017) 151:193–203. doi: 10.1016/j.chest.2016.10.010 27780786

[14] Rami-Porta R Asamura H Travis WD Rusch VW. Lung cancer - major changes in the American joint committee on cancer eighth edition cancer staging manual: The eighth edition of the TNM classification for lung cancer. CA: A Cancer J Clin (2017) 67:138–55. doi: 10.3322/caac.21390 28140453

[15] WHO. Physical status: the use and interpretation of anthropometry. report of a WHO expert committee. World Health Organ Tech Rep Ser (1995) 854:1–452. 8594834

[16] Chen Z Fillmore CM Hammerman PS Kim CF Wong KK. Non-small-cell lung cancers: a heterogeneous set of diseases. Nat Rev Cancer (2014) 14:535–46. doi: 10.1038/nrc3775 PMC5712844 25056707

[17] Jordan KW Adkins CB Su L Halpern EF Mark EJ Christiani DC. Comparison of squamous cell carcinoma and adenocarcinoma of the lung by metabolomic analysis of tissue–serum pairs. Lung Cancer (2010) 68:44–50. doi: 10.1016/j.lungcan.2009.05.012 19559498 PMC2834857

[18] Ding Y Zhang L Guo L Wu C Zhou J Zhou Y. Comparative study on the mutational profile of adenocarcinoma and squamous cell carcinoma predominant histologic subtypes in Chinese non-small cell lung cancer patients. Thorac Cancer (2020) 11:103–12. doi: 10.1111/1759-7714.13208 PMC6938761 31692283

[19] Wang BY Huang JY Chen HC Lin CH Lin SH Hung WH. The comparison between adenocarcinoma and squamous cell carcinoma in lung cancer patients. J Cancer Res Clin Oncol (2020) 146:43–52. doi: 10.1007/s00432-019-03079-8 31705294 PMC11804334

[20] Gonzalez Barcala FJ Garcia Prim JM Moldes Rodriguez M Alvarez Fernandez J Rey Rey MJ Pose Reino A. Platelet count: association with prognosis in lung cancer. Med Oncol (2010) 27:357–62. doi: 10.1007/s12032-009-9217-9 19381892

[21] Yu D Liu B Zhang L Du K. Platelet count predicts prognosis in operable non-small cell lung cancer. Exp Ther Med (2013) 5:1351–4. doi: 10.3892/etm.2013.1003 PMC3671769 23737877

[22] Ji Y Sheng L Du X Qiu G Su D. Elevated platelet count is a strong predictor of poor prognosis in stage I non-small cell lung cancer patients. Platelets (2015) 26:138–42. doi: 10.3109/09537104.2014.888547 24679181

[23] Oncel M Kiyici A Oncel M Sunam GS Sahin E Adam B. Evaluation of platelet indices in lung cancer patients. Asian Pacific J Cancer Prev (2015) 16:7599–602. doi: 10.7314/APJCP.2015.16.17.7599 26625768

[24] Sabrkhany S Kuijpers MJE Oude Egbrink MGA Griffioen AW. Platelets as messengers of early-stage cancer. Cancer Metastasis Rev (2021) 40:563–73. doi: 10.1007/s10555-021-09956-4 PMC8213673 33634328

[25] Haemmerle M Stone RL Menter DG Afshar-Kharghan V Sood AK. The platelet lifeline to cancer: Challenges and opportunities. Cancer Cell (2018) 33:965–83. doi: 10.1016/j.ccell.2018.03.002 PMC5997503 29657130

[26] Budisan L Zanoaga O Braicu C Pirlog R Covaliu B Esanu V. Links between infections, lung cancer, and the immune system. Int J Mol Sci (2021) 22: 9394. doi: 10.3390/ijms22179394 PMC8431665 34502312

